# The Wounded Healer: A Phenomenological Study on Hospital Nurses Who Contracted COVID-19

**DOI:** 10.3389/fpubh.2022.867826

**Published:** 2022-06-30

**Authors:** Michela Piredda, Jacopo Fiorini, Anna Marchetti, Chiara Mastroianni, Beatrice Albanesi, Lucilla Livigni, Gemma Carrabs, Francesco Zaghini, Maria Grazia De Marinis, Alessandro Sili

**Affiliations:** ^1^Research Unit Nursing Science, Campus Bio-Medico di Roma University, Rome, Italy; ^2^Nursing Research Team, Tor Vergata University Hospital, Rome, Italy; ^3^Department of Public Health and Pediatrics, University of Torino, Turin, Italy; ^4^Occupational Health Service, University of Rome Tor Vergata, Rome, Italy

**Keywords:** attitude of health personnel, COVID-19, life change events, nurse, wounded healer

## Abstract

Since the pandemic began nurses were at the forefront of the crisis, assisting countless COVID-19 patients, facing unpreparedness, social and family isolation, and lack of protective equipment. Of all health professionals, nurses were those most frequently infected. Research on healthcare professionals' experience of the pandemic and how it may have influenced their life and work is sparse. No study has focused on the experiences of nurses who contracted COVID-19 and afterwards returned to caring for patients with COVID-19. The purpose of this study was therefore to explore the lived personal and professional experiences of such nurses, and to describe the impact it had on their ways of approaching patients, caring for them, and practicing their profession. A phenomenological study was conducted with 54 nurses, through 20 individual interviews and 4 focus groups. The main finding is that the nurses who contracted COVID-19 became “wounded healers”: they survived and recovered, but remained “wounded” by the experience, and returned to caring for patients as “healers,” with increased compassion and attention to basic needs. Through this life-changing experience they strengthened their ability to build therapeutic relationships with patients and re-discovered fundamental values of nursing. These are some of the ways in which nurses can express most profoundly the ethics of work done well.

## Introduction

The spread of COVID-19, declared a pandemic by the World Health Organization in March 2020, put a strain on all healthcare systems (1). In just a few weeks, the number of hospitalized patients multiplied, dramatically increasing the workload of healthcare professionals (2). Since the beginning nurses were at the forefront of the pandemic scene, assisting countless COVID-19 patients, facing uncertainty, unpreparedness, misinformation and lack of adequate personal protective equipment (PPE) (3) that heavily exposed them to safety risk factors (4). They immediately and repeatedly had to modify teams, activities, procedures and reorganize workplaces. They assisted patients in a new and unpredictable situation (5, 6), engaging in highly invasive procedures (e.g., oro-tracheal intubation to maintain adequate respiration) and end-of-life conversations, and witnessing their patients' isolation from family and friends (6). In this scenario, nurses also had to change their way of approaching patients, providing task-oriented care, and inevitably reducing the time of direct patient care, with a decrease of fundamental nursing care activities such as touch, physical contact, and non-verbal communication (7).

Although the World Health Organization and many scientific associations have published guidelines and recommendations to reduce the risk of COVID-19 transmission among health professionals (8, 9), over the course of the pandemic cases of positivity increased, and healthcare systems and organizations also faced a personnel shortage. Although data on infection and mortality are not internationally homogeneous, the COVID-19 infection rate in healthcare professionals has been estimated to be between 3 and 29% (10) and mortality at one out of every 100 positive (11). Nurses were the healthcare workers most frequently infected (48%), followed by physicians (25%) -who recorded a higher death rate (11, 12) and by other healthcare workers (23%) (4, 13).

In 2 years of the pandemic, research on the effects of COVID-19 on health professionals has focused on the need for supporting them (14) and on identifying interventions introduced to limit the impact of the pandemic on organizational wellbeing, such as redistribution of workloads, reorganization of care models, control of burnout, training on the use of personal protective equipment, health surveillance and the establishment of psychological support desks (15–18). There has also been a focus on nurses' physical and psychological health, in terms of stress, anxiety, post-traumatic stress disorder and insomnia (15, 19–23).

However, few studies have evaluated healthcare professionals' experience of this pandemic and how it may have influenced their life and work. The qualitative and mixed-method studies conducted on nurses' experiences at the time of the pandemic found a number of issues and emotions felt in facing the unknown and in the care of complex patients with new needs (18, 24, 25). Nurses reported that they were afraid of contracting the virus and exposing their family members to it, that they were stigmatized by society as a source of infection, that they believed they had not received enough support from organizations, and that they needed psychological and spiritual support for themselves and for patients (24, 26–28). Specifically, only two studies explored the experience of nurses who contracted the virus, but they were limited to the hospital isolation (29) or quarantine and treatment period (30).

No study has been found that focused on the experiences of hospital staff nurses who, after contracting COVID-19, returned to caring for patients with COVID-19. These experiences can have a different impact on the personal and professional lives of nurses comparing with those during the illness or those of nurses who did not become ill.

Therefore, the purpose of the present study was to explore the lived personal and professional experiences of hospital nurses in Italy who, after contracting COVID-19, returned to caring for patients with COVID-19, to elucidate the impact it had on their way of approaching patients, caring for them, and practicing their profession.

## Materials and Methods

### Study Design

A qualitative study was conducted with a descriptive phenomenological approach based on Husserl's (31) life world perspective, in order to understand in depth the lived experiences of nurses who contracted COVID-19, from an individual, social and occupational point of view (32, 33). Phenomenology deals with the phenomenon of consciousness, as regards to the totality of lived experiences belonging to a single person (31). It focuses on the existential meaning that is common to all people who have the experience (34). The descriptive method of Giorgi (35) was used, which is based on 5 steps: data collection, data reading, data division into sub-parts, organization and reprocessing of data in ordinary, comprehensible language for dissemination, and describing the structure of the phenomenon. The COREQ checklist was followed for drafting and conducting the study (36).

### Participants

The study was conducted in a 600-beds University Hospital located in a big city in central Italy that in the first wave of the pandemic was entirely dedicated to the care of COVID-19 patients. During the first and the second wave of the pandemic overall 120 nurses (about 7 and 5,6% of total nurses, respectively) contracted COVID-19, either at work or outside the hospital. A purposive sampling method was used to select a heterogeneous sample of nurses with regard to age, sex, years of work, severity of disease, clinical area (e.g., medical, surgical, ICU) and family status (37–39). All nurses who cared for COVID-19 positive patients for at least 2 weeks and had become positive for COVID-19 were considered. Their names were gathered through the internal organization notification system of the nurses who became positive for COVID-19 as taken into care by the Hospital' s Occupational Medicine. A letter was sent to each of these nurses giving information about the study and asking their consent to participate.

### Data Collection and Instruments

Data collection lasted from March to June 2021. First, 20 individual interviews were conducted from March to April 2021; then, 4 focus groups were held between May and June 2021 with 34 nurses who had not been interviewed previously.

A topic interview guide was developed, piloted with the first five participants (three women and two men), and then used to also conduct the remaining interviews. It included open-ended questions focused on the subjective experiences of nurses who had become COVID-19 positive patients, to explore in-depth the perceived emotional, cognitive and relational meaning of being ill, and impact the disease had on daily life (family relationships, home organization) and on the relationships with work organization, colleagues and patients, when returning to work. Examples of questions were the following: How did you live through the experience of being positive for COVID-19 (perceptions, feelings, thoughts)? What did it mean for you to be COVID-19 positive? What kind of impact has your illness had from an emotional, cognitive, relational point of view? What do you think has changed in your life and your relationship with others (family, friends, patients) after this experience? How did you feel about returning to a relationship with patients (positive or not) after your illness? Four focus groups were then held using a similar topic guide, to further explore the personal themes that crossed the different interviews, to identify common experiences and interactions with colleagues, patients, family and the impact of COVID-19 on their life.

Individual in-depth interviews and focus group discussions were conducted in Italian by four researchers who were experts in qualitative interviewing and focus groups moderation. Each interview and focus group was conducted by two researchers, one acting as interviewer/moderator and the other as observer and assistant, welcoming participants, collecting signed consent forms and socio-demographic data, audio-recording, and taking field notes including relational dynamics, interpersonal climate, and non-verbal communication. The researchers created a warm, non-threatening atmosphere, giving participants the confidence to answer as spontaneously and truthfully as possible. The focus groups and interviews were held in reserved spaces without potential noise distractions, digitally recorded, transcribed, and integrated with the field notes. The focus groups followed an adapted version of the interview topic guide, to address the issues that had emerged in the interviews while capitalizing on the group discussion to identify common experiences.

### Data Analysis

Data were analyzed following the steps of Giorgi's phenomenological descriptive approach (35): (1) interviews transcription and reading (they were read several times, to understand the experiences of COVID-19 positive nurses in depth); (2) data subdivision into sub-parts; (3) organization and linguistic re-phrasing of data; (4) describing the structure of the phenomenon. After the first reading of the data, the research team focused on the text as a whole, not analyzing the thematic aspects of the phenomenon, but highlighting the general sense of the entire situation experienced by COVID-19 positive nurses. In the second step, the subdivision of the data into sub-parts, analysis of the text for each participant was carried out, marking the different units of meaning—small parts of the text with meaning relevant to the study expressed in the language of the participant—and further clarifying them linguistically. In the third step, organization and linguistic re-phrasing of the data, the units of meaning were re-described to express their explicit values, standardizing them in ordinary, comprehensible language. In the last step, describing the structure of the phenomenon, a similar procedure was followed, and the units of meaning were transformed with the help of free imaginative variation. Free imaginative variation is the intellectual process by which researchers consider different examples of a phenomenon to discover its constant and essential elements. The purpose of this process was to determine which unit was essential for the phenomenon analyzed, and to describe the essential structure of the lived experience from the perspective of healthcare professionals, first as professionals and then as patients. Given that the outcome of phenomenological analysis is not the essential structure but how the structures relate to the various manifestations of an essential identity (35), the structures understood as essences and their relationships were made explicit, leading researchers to uncover the themes, subthemes and general structure described below.

### Ethical Considerations

The study was conducted in accordance with the Helsinki principles (40) and was approved by the Ethics Committee of the hospital where the study was conducted (Protocol No. 80/20). Eligible nurses were informed about the study aims and procedures; it was explained that participation was voluntary, and that they could withdraw at any time. They were also informed that their data would be treated confidentially, and their identities would not appear in written records. Willing participants signed an informed consent to study participation and to data treatment in accordance with current law regarding privacy. The individual interviews and the focus groups were conducted in a quiet, dedicated room, which guaranteed full privacy. The data were collected, processed, and analyzed in compliance with privacy and anonymity: in the transcription phase, participants' names were replaced by alphanumeric codes.

### Rigor

Following the principles of qualitative research (41, 42), the research team worked to ensure the rigor of the study on five criteria: credibility (strategies implemented to ensure the credibility of results), dependability (use of approaches designed to ensure the replicability of the results), confirmability (ensuring that the results faithfully represent the participants' narratives), transferability (degree of agreement of the results of a study with other settings similar to the study area) and authenticity (providing details of the descriptions of the participants' experiences and feelings experienced by the participants in relation to the phenomenon studied). These criteria were ensured in the study by writing out the method in detail, the use of a topic guide for focus groups and interviews, transcription of notes, recording of meetings, phase analysis, and the enrolment of nurses from varied socio-demographic and clinical backgrounds. Moreover, only the meaning units that appeared with a reasonable frequency in different interviews and groups were included. Most words were repeated 5–10 times each or more.

## Results

Fifty-four nurses, 39 female and 15 male, with a mean age of 43.6 ± 7.5 (range 30–57) years and mean work experience of 11.5 ± 6.6 (range 1–19) years, participated in this study: 20 in the individual interviews and 34 in the 4 focus groups. They were COVID-positive for a mean of 30.9 ± 13.6 (range 7–58) days. Most nurses quarantined at home (*n* = 44), and only ten were hospitalized for a mean of 17.8 ± 15.9 (range 2–46) days. Participants in each focus group ranged between 7 and 10 nurses (total 34, 23 female and 9 male).

The findings include the general structure and two themes with six and three subthemes, respectively, which will be presented below with excerpts from the transcripts to support them. The sources will be identified with alphanumeric codes for the individual interviews (IN1–20) and for the group discussions (FG1–4).

### General Structure: The Wounded Healer

The overarching theme that emerged from the findings is that of the “wounded healer,” who is a nurse who contracted COVID-19, survived and recovered, but remained “wounded” by the experience and returned to caring for patients as a “healer,” with increased compassion and attention to basic needs.

#### Theme 1: Wounded: Contracting COVID-19 Generates Profound Personal and Social Changes

##### Traumatic Experience

Nurse participants described their experience as a traumatic and strongly negative one. For instance, they said: “*It was devastating, a deep chasm with no way out” (IN8), “It was a calvary” (FG1), “We lived it as a tragedy, because we were the first and we did not know how it would end, and how to manage it” (IN8)*, and “*It was an emotional and physical trauma”*. They reported unpleasant emotions such as anger, meaninglessness, fear, sadness, tiredness, worry, anxiety, and guilt. For instance, one participant noted: “*I sank into this meaningless situation”*. Another shared: “*The fear: shall we come back to normal life? (FG3)”*. Interestingly, one nurse voiced her experience as follows: “*I felt discomfort due to the broken relationship between me and my body” (IN6)*, and another said: “*Our energies ran out (…) mine ran out to the point of being extinguished” (IN20)*. Fear, anxiety and worry were experienced not only for their own lives, but also, especially, for other people's lives. They were afraid of infecting their family, colleagues, or weak patients. For instance, they said: “*As long as you are single, you worry less” (IN18);* and also: “*As a daughter (you feel) the fear of harming your mother, and as a mother (you feel) the fear of harming your daughter” (IN19)*. Similarly, they felt guilty when they infected their family members or patients and expressed their sadness at “*being cause of the suffering of others” (IN18)*, and also: “*You feel guilty if the patient is positive” (FG3)*, and as another put it: “*this (feeling guilty about my daughters) floored me, it did not knock me out completely but it did floor me” (IN9)*.

##### Revelation of One's Own Vulnerability

Nurses expressed becoming aware of not being immune, feeling defenseless, powerless, and inadequate to face the situation. For instance, one participant noted: “*It made all of us vulnerable, including those who had felt powerful before” (IN6)*. Feelings of powerlessness and lack of control were described by saying: “*I am trying to mend something that got out of my control” (IN11)*, and using the images of being like “*a boat overwhelmed by a storm or a crazy ball in a pinball machine” (FG4)*. They felt “*powerless by depending on the help of others (FG1)* and “*humiliated because depending on other (to bring them the meals)” (FG2)*. Loss of freedom was also reported as: “*I felt like a butterfly without wings” (IN9)*. They seem to have discovered that in this “*unpredictable situation” (FG4)* they “*are not as essential as in the beginning” (IN20)* to solve it, and that “*on getting to a certain point we must surrender” (IN14)*.

##### Impact on Family Life

Participants reported the impact of their disease on family life, particularly on young children, as a hard experience. For instance, they said: “*The worst sensation: leaving my little baby and not knowing what was waiting for me” (IN18); “All the children were crying, full of fear” (IN8) and “They said: Mum, please, get better and change your job, I don't like your job” (FG3)*. The need for physical contact with loved ones was clear both for children and adults: “*It was devastating for the children to stay physically far from their mother” (FG1), “Children cannot live in social isolation” (IN6); “My husband sent me a heart-shaped pillow. I hugged it in that hospital bed, as if all my family were there with me” (IN8)*. They wanted to protect their family and were worried about the possibility of infecting them. Thus, when they infected some or all of their families, they felt guilty: “*I did not think of myself at all, I was not focused on myself, but on my family,” “I worried about my family: I infected my 2-year-old daughter, and my pregnant wife” (FG3)*.

When isolation was lived at home together with the family, sometimes it became an opportunity for sharing life in a deeper way than ever: “*Staying at home for one month will never happen again, and I lived it to the full. We managed time together, the four of us in 50 square meters of space. I made the most of it, actively and enjoyably. I had many things to do that could be done at home, and they (children) were in DAD (remote school classes). Actually, for a month I “detoxed.” I looked at the positive side of the event, living it as a new experience for me and my family. It united us.” (FG1)* And another nurse noted: “*We all became (Covid) positive (in my family): the paradoxical happiness of being able to hug each other and stay together” (FG3)*.

##### Isolation

Participants reported having experienced physical and social isolation (in the family, in the hospital, among colleagues, and in their neighborhood). In many instances they spoke about loneliness, isolation (which was also referred to as a protection) and being confined in the home. A nurse noted: “*I felt I was all alone, just me and the virus” (IN8)*. And another said “*This Covid has opened up this issue, of leaving people alone and thinking that they could manage” (IN20)*. Isolation was experienced as being enclosed in a room or house: “*I felt shut in, under house arrest” (FG4), “now that I too have been shut in a room, far away from loved ones…” (FG3)*. A particularly terrible experience was reported by a nurse who was put in sealed bio-containment, as being “*like in a coffin, a niche” (IN8)*. Nurses noted strongly how they missed physical contact. For instance, they reported: “*How important a hug is, what a consolation” (IN8)*. They recognized the importance of physical contact for patients too by saying: “*Lack of contact in hospitals, in my view, is something that just isn't fair” (IN6)*; and also: “*It is traumatic that now I cannot touch them” (IN15)*.

##### Conflicting Social Image of the Nurse

This theme describes how nurses perceived the impact that COVID-19 has had on the social image of nurses. Nurses reported that, on the one hand, society has praised them and described them as heroes and angels, making them feel trustworthy custodians of health; on the other hand, it has considered them as sources of infection, leading to perceived abandonment and ingratitude by society. For instance, they shared: “*Someone who praised you and described you as a hero and angel, later accuses you of being an infector and lacks gratitude toward you” (FG3); “We experienced the hero moment, but then if you are a nurse, they give you a dirty look (FG4)”; “First, heroes who save Italy, the nurse with Italy in her arms: “We trust you”... but then we are abandoned” (IN20)*.

##### Sense of Injustice, Abandonment and Solidarity

Feelings of injustice were voiced, in particular because of lack of organization and resources, such as PPE: “*They were not able to manage some situations” (IN20), “I am disappointed by the injustice” (IN14), “They asked too much of us” (IN20), “Access to care became impossible” (IN6)*. In many cases the loneliness caused by isolation generated experiences of abandonment, by society, healthcare services in the community and the organization they belong to. They reported: “*As soon as you become COVID-19 positive you are abandoned to yourself” (FG3), “I felt I was not being supported” (IN9), “A patient cannot be abandoned in these conditions” (IN4)*. For others, by contrast, it gave rise to experiences of solidarity and closeness (from neighbors, colleagues, superiors, the organization, etc.).

#### Theme 2: The Healer: Rediscovering the Origins of Nursing Profession and Its Founding Values

This theme describes how the experience of contracting COVID-19 has helped nurses rediscover important aspects of nursing and the founding values of their profession.

##### New Knowledge for the Management of Basic Care Needs

Nurse participants reported having gained more knowledge and preparation on how to manage the care needs of patients with COVID-19 in terms of priorities, methods, and times of the procedures related to basic care needs: “*In the minutes it takes to brush the patient's teeth, the patient desaturates, therefore you really have to hurry… you can see him struggling. (…) Many patients have blood thinning and the oral cavity must be well cared for otherwise it will bleed” (IN18); “You change the diaper, make them drink, feed them, dry the secretions, find a suitable pillow, move the helmet, fix the tube. If you ensure their comfort, they may be able to overcome the disease” (IN19); “It may seem trivial, but you have to be careful: you have to think before you do anything” (IN3)*.

##### A Deeper Understanding of the Patient's Needs

Nurses noted that they have acquired a deeper understanding of patients' and their family members' needs thanks to a process of identification that led to a change in the care relationship: “*I remember when I was on the other side. You see in that person what you have already experienced, and you identify yourself more with their sufferings” (FG2); “Now I also take care of the little things that give joy because they are the ones that I missed the most” (IN8)*.

Participants reported feeling more involved in the relationship with the patient and showing more empathy toward the communicative and emotional needs of the patient. “*Now it is difficult to remain detached, I understand patients better and I get more in tune” (FG1); “Personal experience has allowed me to increase that degree of empathy that has always been a part of me” (FG2); “They are isolated, they have no contact with the outside world and you try to put them in communication by making video calls to relatives” (FG1); “Patients stay months in bed, nobody sees them... there is a need for humanity (IN19)”; “I am now much warmer toward those who are sick, I feel like cuddling them” (IN8)*.

##### Rediscovering the Bond With Colleagues and the Founding Values of the Nursing Profession

The nurses shared that the perception of the closeness, support, and solidarity of colleagues strengthened the bond between them: “*I have greatly strengthened the bond with colleagues as sisters” (FG4); “My colleagues have always been close to me: I expressed my thoughts only to them”; “Fantastic colleagues, we supported and backed up each other, respecting those who needed to be left alone...” (IN6); “I received a solidarity that I did not expect” (IN10); “Their unexpected affection has enriched me” (IN4)*. Finally, nurses claim that they have rediscovered the value of their profession and what motivates them to exercise it: “*I have rediscovered the true value of this profession (…) I have recovered the enthusiasm of the beginnings for my profession” (FG4); “I feel like a new nurse” (IN8); “As soon as I had the chance I ran back to work, because it is my strength” (IN9)*.

## Discussion

This study aimed at uncovering the lived personal and professional experiences of nurses who, after being affected by COVID-19, returned to patient care. The main finding of the study is that the nurses who contracted COVID-19 became “wounded healers”: they recovered but remained “wounded” by the illness experience and returned to caring as “healers” with increased compassion, empathy, and attention to patients' fundamental needs. The nurses' experience of being ill with COVID-19 changed them profoundly, both personally and professionally. It influenced their way of perceiving life and social relationships. It showed them their human vulnerability and powerlessness. Having suffered from the same illness provided them with a much deeper understanding of their patients' and families' experiences and needs. Through this life-changing experience, they gained new knowledge, strengthened their ability to empathize and re-discovered fundamental values of nursing.

The archetype of the wounded healer is rooted in the Greek myth of Chiron. The immortal centaur Chiron was wounded by Heracles' arrow and suffered unbearable pain for the rest of his life. He was able to transcend and transform his suffering in order to heal others, becoming a legendary healer. Because of his wound he gained transformative characteristics that are crucial to help the healing of others (43). The term “wounded healer” as such was first used by Jung who believed that only a “wounded” physician could heal effectively (44, 45). The notion of wounded healer, generated in the field of psychiatry and psychotherapy, expanded to include any helping profession including nurses, who involve their unique personal characteristics when addressing the needs of vulnerable people (46).

Marion Conti-O' Hare (47) developed the theory of the “Nurse as Wounded Healer” with the tenet that individuals exposed to personal trauma can develop either effective or ineffective coping strategies. Individuals with ineffective coping, whose trauma is not recognized and whose pain is unresolved, may act as “walking wounded,” projecting their conflicts on patients and colleagues and being less able to show empathy with others. By contrast, individuals who deal effectively with the trauma are able to recognize, transcend and transform their pain into healing. Although the “scar” remains, personal injuries that have been the object of deep reflection not only will not undermine care provision, but can lead nurses to become “wounded healers,” by improving their ability to build therapeutic relationships with patients (47). It is not only their suffering that transforms them into healers but also the awareness of their woundedness and their willingness to accept and transcend it, by integrating it into their relationship with their patients.

Nurses who were affected by COVID-19 could take advantage of their own experience of suffering, powerlessness, vulnerability and needing more care than they received, to improve their care for patients. On crossing into the world of patients and finding themselves at the mercy of the illness, not immune but care dependent, nurses seemed unprepared for the feelings of powerlessness associated with contracting COVID-19, and experienced something like a “shock of becoming a patient” (18, 48). Indeed, nurses' vulnerability may be different and greater than for other patients, as nurses are more used to giving care and less used to receiving it. Moreover, they have greater knowledge, and therefore perhaps also greater expectations, and feel the need to stay in control of the care process (48–50). However, these nurses were able to learn from their experience through analysis and reflection, which enabled them to fill their relationships with patients with therapeutic content. Nurses reacted with greater empathy and a greater ability to identify themselves with patients, and that enabled them to better personalize their care to the needs of each patient, a care that they had sometimes missed when they were sick, and that they would have wished for themselves. They were no longer in an asymmetric or paternalistic relationship with patients (51). These nurses came back to patient care more aware of being part of the same vulnerable and mortal humanity as the patients they were caring for. This mindfulness of their own fragility, brokenness and connectedness to others enabled them to be companions and healers of others who were suffering (52). As Conti-O' Hare (47) put it: “When people who have developed wounding gain sound insights into their own situations they are in a better position to communicate human warmth, which in turns helps patients heal” (p. 2). This ability to build therapeutic relationships with patients is one of the most profound ways in which nurses can express the ethics of work done well (53–55).

Contracting COVID-19 generated profound personal and social changes in nurse participants. They described it as a highly traumatic experience, reported feeling vulnerable because of their direct contact with COVID-19 patients, expressed concerns about their health and that of their family, and awareness of being a possible source of infection for their family members, in line with previous studies conducted with healthcare professionals involved in the frontline during the pandemic (5, 56–58).

The experience of isolation was also reported as strongly impacting nurses' feelings. Social and physical isolation was perceived as something unfair and terrible for human beings, especially for vulnerable people such as patients and children. This is also in line with previous research (59, 60), describing how exposure to biological risks, such as the pandemic, could be a major source of stress for nurses, leading them to isolate themselves physically from their family members, to protect them from possible contamination (14).

Being COVID-19 positive also had a profound impact on nurses because of the social image of the nurse who at the beginning was perceived as a hero, and afterwards as a source of infection associated with stigma. This conflicting image has also been described by Alsaqri et al. (61) in a qualitative work reporting how nurses diagnosed with COVID-19 found themselves being stigmatized both in their workstation and the community, during quarantine and even after complete recovery.

Participants complained of feeling abandoned by healthcare organizations, community and society, and reported feeling a profound sense of injustice about this because they were infected while serving at the frontline of a dangerous pandemic. This accords with similar reports of unsupportive environments (61). However, nurses in this study also reported numerous offers of presence and solidarity from colleagues, superiors and other members of their healthcare teams that made them feel accompanied and thought about during their isolation period. This represented a great solace during those difficult times, and in most cases facilitated their return to caring for patients with COVID-19 within the same team.

Because of the process of reflection, transformation and transcendence of their own illness experience, nurses became able to rediscover the founding values of nursing. This was shown, in particular, by their gaining a new understanding and knowledge of the importance of little things when attending to basic patient needs (7), personalizing care and strengthening the bond with colleagues.

Although generalization is not a goal for qualitative studies, we used strategies able to warrant transferability of results to other settings. However, the study was conducted in a single center with self-selected participants who therefore may not be representative of all nurses who recovered from COVID-19 and came back to care for patients with COVID-19.

## Conclusion

Findings from this study are highly relevant to nursing and should inform nursing hospital management and education. Each human life is exposed to traumas, over and beyond COVID-19. Nurse managers and educators should be aware that for “wounded” nurses not to remain “walking wounded,” but to take the path of becoming “wounded healers,” traumatic experiences need to be interiorized and transformed. To help this process, managers should foster significant relationships with nurses enabling them to engage in trusting discussions, to reflect on their own experiences and transform them into “living material” with the potential of becoming a personal life project, able to orient care activities. Similarly, educators should develop educational paths in which students are stimulated to learn from traumatic experiences and become wounded healers. To this end educational settings must be student-centered, and prioritize trusting relationships with students, enabling them to share their experiences and look for help to reflect on and rework them. These are not simple or common tasks. They require nurse managers and educators being prepared to help nurses and students in this transformational reflective path. The COVID-19 pandemic has offered a chance to rediscover and rethink the nursing profession, especially through the priority attached to basic patient care. As each crisis can represent an opportunity, becoming wounded healers has shown great potential for promoting the personal and professional growth of nurses that can also result in “well done care work” (53). We must capitalize on these lessons learned and use them to produce rich fruits in present and future generations of nurses.

## Data Availability Statement

The raw data supporting the conclusions of this article will be made available by the authors, without undue reservation.

## Ethics Statement

The studies involving human participants were reviewed and approved by Protocol No. 80/20. The patients/participants provided their written informed consent to participate in this study. Written informed consent was obtained from the individual(s) for the publication of any potentially identifiable images or data included in this article.

## Author Contributions

MP: conceptualization, methodology, investigation, formal analysis, data curation, visualization, and writing—original draft. JF: conceptualization, methodology, visualization, writing—original draft, and writing—review and editing. CM: investigation and formal analysis. BA and AM: conceptualization, investigation, formal analysis, and writing—original draft. LL and GC: conceptualization, investigation, and formal analysis. FZ: conceptualization and writing—original draft. MD: conceptualization, writing—review and editing, and supervision. AS: conceptualization, methodology, writing—original draft, writing—review and editing, and supervision. All authors contributed to the article and approved the submitted version.

## Conflict of Interest

The authors declare that the research was conducted in the absence of any commercial or financial relationships that could be construed as a potential conflict of interest.

## Publisher's Note

All claims expressed in this article are solely those of the authors and do not necessarily represent those of their affiliated organizations, or those of the publisher, the editors and the reviewers. Any product that may be evaluated in this article, or claim that may be made by its manufacturer, is not guaranteed or endorsed by the publisher.
